# Symmetric dimethylguanidino valeric acid, a novel single biomarker of hepatic steatosis

**DOI:** 10.1016/j.isci.2024.111366

**Published:** 2024-11-13

**Authors:** Roman N. Rodionov, Natalia Jarzebska, Yen Chin Koay, Mengbo Li, Matthias Kuhn, Stefan R. Bornstein, Jens Martens-Lobenhoffer, Mohammad Eslam, Fei Wen Chen, Elena Rubets, Alexander G. Markov, Norbert Weiss, Andreas Birkenfeld, Peter Schwarz, Stefanie M. Bode-Böger, Nikolaos Perakakis, John F. O’Sullivan, Jacob George

**Affiliations:** 1 Department of Internal Medicine III, University Center for Vascular Medicine, Technische Universität Dresden, 01307 Dresden, Germany; 2 College of Medicine and Public Health, Flinders University and Flinders Medical Centre, Adelaide, SA 5042 Australia; 3 Department of Anaesthesiology and Intensive Care Medicine, University Hospital Carl Gustav Carus, Technische Universität Dresden, 01307 Dresden, Germany; 4 Cardiometabolic Medicine, School of Medical Sciences, Faculty of Medicine and Health, The University of Sydney, Camperdown, NSW, Australia; 5 Charles Perkins Centre, The University of Sydney, Sydney, NSW, Australia; 6 Bioinformatics Division, The Walter and Eliza Hall Institute of Medical Research, 1G Royal Parade, Parkville, VIC 3052, Australia; 7 Department of Medical Biology, The University of Melbourne, Parkville, VIC 3010, Australia; 8 Institute for Medical Informatics and Biometry, Faculty of Medicine Carl Gustav Carus, Technische Universität Dresden, Dresden, Germany; 9 German Center for Diabetes Research (DZD e.V.), Ingolstädter Landstrasse 1, 85764 Neuherberg, Germany; 10 Department of Internal Medicine IV, Department of Endocrinology, Diabetology and Nephrology, University Hospital of Eberhard-Karls-University Tübingen, Tübingen, Germany; 11 Germany and Institute for Diabetes Research and Metabolic Diseases of the Helmholtz Center Munich at the Eberhard-Karls University of Tübingen, 72074 Tübingen, Germany; 12 Department of Diabetes, School of Life Course Science and Medicine, King’s College London, London, UK; 13 Paul Langerhans Institute Dresden (PLID), Helmholtz Center Munich, University Hospital and Faculty of Medicine, TU Dresden, Dresden, Germany; 14 Institute of Clinical Pharmacology, Otto-von-Guericke University, Magdeburg, Germany; 15 Storr Liver Centre, Westmead Institute for Medical Research, Westmead Hospital and University of Sydney, Sydney, NSW, Australia; 16 Department of General Physiology, St. Petersburg State University, St. Petersburg, Russia; 17 Department of Cardiology, Royal Price Alfred Hospital, Sydney, NSW, Australia

**Keywords:** Health sciences, Clinical finding, Physiological state

## Abstract

There is an unmet need for a biomarker of liver fat. We identified dimethylguanidino valeric acid (DMGV) as a circulating biomarker of liver fat. Here, we assess its two isoforms—symmetric (SDGV) and asymmetric (ADGV)—as biomarkers of steatosis. We determined plasma ADGV, SDGV, related metabolites, alanine aminotransferase (ALT), and the fatty liver index (FLI) in two cohorts and compared their diagnostic performance for liver fat detection. SDGV was the strongest predictor of moderate to severe steatosis. Changes in SDGV correlated with changes in liver fat % in a prospective cohort. In a murine model of fatty liver disease, protein levels and activity of alanine:glyoxylate aminotransferase 2 (AGXT2), which produces SDGV, were increased and coincided with elevation of SDGV concentrations. SDGV is a biomarker of liver fat and its increase in hepatic steatosis results from the upregulation of AGXT2 activity.

## Highlights

• Plasma levels of SDGV are equal to, or more discriminatory for liver fat than the FLI
• Elevated levels of SDGV result from increased enzymatic activity of AGXT2
• Our findings have the potential to improve detection of NAFLD in clinical practice

## Graphical abstract

**Figure:**
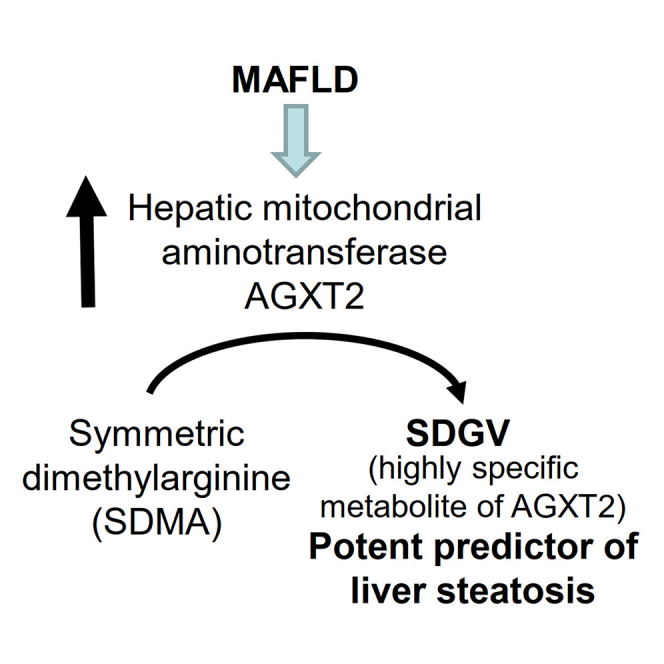

## Introduction

Fatty liver disease due to metabolic dysregulation is driven by the pandemics of overweight/obesity and diabetes. In the USA, one-third of the adult population and 10–20% of children suffer from the disease. The prevalence in Europe and Asia is slightly lower, but still affects about 25% of adults.^1^ Risk factors include, but are not limited to, central obesity, type 2 diabetes, and dyslipidemia.^2^ The early stages of this disease are characterized by liver fat accumulation without inflammation; however in 5–10% of individuals, a fatty liver can progress to steatohepatitis, which can lead to fibrosis, cirrhosis, or to the development of liver cancer.

In managing patients with fatty liver disease, there is firstly a need to diagnose the presence of liver fat, and secondly a need to stage disease activity and fibrosis. This manuscript focusses on the first problem, liver fat. Currently, there is no simple blood-based biomarker to assess for the presence of liver fat, to quantify its extent or to monitor its presence over time. The gold standard for staging fatty liver disease, including an assessment of liver fat, is liver biopsy. However, biopsy is an invasive and costly method that carries risks for the patient and is not optimal for monitoring over time. Consequently, there is an urgent need for non-invasive diagnostic tests both to assess for the presence of liver fat and for serial monitoring. In this context, liver ultrasound is most commonly used for diagnosing hepatic steatosis. However, ultrasound has low sensitivity when liver fat content is less than 20%,^3^ is operator-dependent, semiquantitiave, and less useful for longitudinal assessment. The fatty liver index (FLI), developed by Bedogni et al. in 2006, is an algorithm that can predict the presence of steatosis based on body mass index, waist circumference, triglyceride, and gamma glutamyltransferase levels that has been used for clinical research. However, the FLI is rarely used in daily clinical practice either in primary care or specialty practice.

Alanine aminotransferase (ALT) is the most sensitive blood test of hepatic necrosis currently available, followed by aspartate aminotransferase (AST).^4^ ALT is able to detect minor liver injury, particularly when using normal healthy values as opposed to reference ranges. Although it is not a marker of fat accumulation, it is nevertheless, along with AST, usually a first step in screening for potential fatty liver disease in primary care.

We recently identified dimethylguanidino valeric acid (DMGV) as a novel circulating biomarker of liver fat.^5^ In this study, we aimed to assess which of its two isoforms—symmetric (SDGV) and/or asymmetric (ADGV)—is responsible for its utility as a biomarker of liver fat. To determine the relationship of liver fat accumulation to the expression of the generative pathway for SGDV and ADGV, we used the *db/db* leptin receptor-deficient mouse, one of the most common and well-established murine models of diabetes mellitus.^6^ Along with obesity, the model demonstrates all the key biochemical and physiological features of metabolic abnormalities associated with liver fat accumulation including fasting hyperglycaemia, hyperinsulinaemia, dyslipidaemia, and micro-and macrovesicular steatosis.^7^

In this study, we (1) determined plasma SDGV, related metabolites, ALT, and the FLI in a large cohort and compared its diagnostic performance for liver fat detection both cross-sectionally and prospectively, (2) we confirmed the importance of DMGV in a second cohort, and (3) investigated the involvement of AGXT2 and its metabolites in a mouse model of fatty liver disease.

## Results

### Baseline characteristics of the cohorts

The characteristics of participants of the Prediabetes Lifestyle Intervenion Study (PLIS) at baseline and one-year after lifestyle intervention and the characteristics of the Sydney-based cohort are listed in Table 1 and Table 2, respectively. In both cohorts the majority were men (62% in PLIS and 61% in Sydney cohort). The mean age was 65 years (PLIS) and 50 years (Sydney) and the mean body-mass index was 28.4 kg/m^2^(PLIS) and 29.1 kg/m^2^ (Sydney). The mean ALT levels were 35.9 IU/L (PLIS) and 72.6 UI/L (Sydney). In the PLIS cohort, the mean intrahepatic liver content was 15.2% (by MRI), and in the Sydney cohort, 66% of participants had moderate to severe (S2-S3) steatosis by histology.

**Table 1:** PLIS cohort characteristics

Variable	Baseline	Year 1
**Women/men, n (%)**	81/132 (38/62%)	81/132 (38/62%)
**Age [years]**	65 ± 8	66 ± 8
**Body Mass Index [kg/m**^**2**^**]**	28.43 ± 4.24	28.63 ± 4.76
**Waist-to-hip ratio [cm/cm]**	0.92 ± 0.08	0.93 ± 0.09
**Visceral fat mass**_**MRI**_**[l]**	5.73 ± 2.41	5.37 ± 2.22
**Liver fat content**_**MRI**_**[%]**	15.21 ± 8.51	12.55 ± 6.34
Fasting glucose [mmol/L]	6.1 ± 0.4	5.9 ± 0.5
**ALT [IU/L]**	35.94 ± 26.64	32.41 ± 18.22
**AST [IU/L]**	29.91 ± 19.03	29.18 ± 17.60
**GGT [IU/L]**	45.70 ± 57.17	40.71 ± 61.65
**HbA1c [mmol/mol]**	39.94 ± 3.16	38.65 ± 4.12
**2-h glucose [mmol/L]**	7.8 ± 1.8	6.9 ± 1.8
**Fasting insulin [pmol/L]**	107.1 ± 63.1	121.9 ± 209.9
**2-h insulin [pmol/L]**	575.9 ± 467.1	638.0 ± 600.0
**Triglycerides [mmol/L]**	1.4 ± 0.9	1.4 ± 0.9
**Total cholesterol [mmol/L]**	5.42 ± 1.05	5.43 ± 1.15
**HDL-cholesterol [mmol/L]**	1.52 ± 0.61	1.40 ± 0.36
**LDL-cholesterol [mmol/L]**	3.07 ± 0.82	3.16 ± 0.95
**HOMA-IR**	4.71 ± 2.97	4.77 ± 8.44
**Hypertension**	86/132 (65%)	76/132 (58%)
**Hyperlipidemia**	46/132 (35%)	46/132 (35%)
**Diabetes type II**	0/132 (0%)	2/132 (1.5%)

Mean ± Standard Deviations are reported. IU/L: international units/Liter; mmol/L: millimoles/liter; pmol/L: picomoles/liter; MRI: magnetic resonance imaging; ALT: alanine aminotransferase; AST: aspartate aminotransferase; GGT: gamma glutamyl-transpeptidase; HbA1c: glycated hemoglobin; HDL: high-density lipoprotein; LDL: low-density lipoprotein; HOMA-IR: homeostatic model assessment of insulin resistance.

**Table 2:** Sydney-based NAFLD cohort characteristics

Variable	Value
**Age, yrs**	50 ± 13
**Male n (%)**	107 (61)
**BMI, kg/m**^**2**^	29.12 ± 14.73
**ALT [IU/L]**	72.62 ± 13.44
**AST [IU/L]**	52.04 ± 15.56
**GGT [IU/L]**	143.09 ± 174.53
**Triglycerides [mmol/L]**	1.9 ± 1.05
**Total cholesterol [mmol/L]**	5.13 ± 2.4
**HDL-cholesterol [mmol/L]**	1.27 ± 0.43
**LDL-cholesterol [mmol/L]**	3.06 ± 1.05
**HOMA-IR**	4.52 ± 1.07
STEATOSIS	–
**None and mild, n (S0-S1) (%)**	66 (34.2%)
**Moderate and severe, n (S2-S3) (%)**	127 (65.8%)
HEPATOCYTE BALLOONING	–
**None and mild (0–1), n (%)**	101 (52.3%)
**Severe (2), n (%)**	92 (47.7%)
**NAS**	–
**≥4, n (%)**	165 (85.5%)
**≤4, n (%)**	28 (14.5%)
FIBROSIS SCORE	–
**F0-1**	78 (40.4%)
**F2-4**	115 (59.6%)
METABOLIC COMORBIDITIES	–
**Hypertension, n (%)**	47 (24.4%)
**Diabetes mellitus, n (%)**	34 (17.6%)
**Dyslipidemia, n (%)**	73 (37.8%)

Mean ± Standard Deviations are reported; BMI: body-mass index; IU/L: international units/Liter; mmol/L: millimoles/Liter; ALT: alanine aminotransferase; aspartate aminotransferase; GGT: gamma glutamyl-transpeptidase; HDL: high-density lipoprotein; LDL: low-density lipoprotein; HOMA-IR: homeostatic model assessment of insulin resistance; NAS: NAFLD (non-alcoholic fatty liver disease) activity score.

### SDGV and ADGV predict liver fat percentage and moderate-severe steatosis in two independent cohorts

#### The PLIS cohort

**Figure 1:**
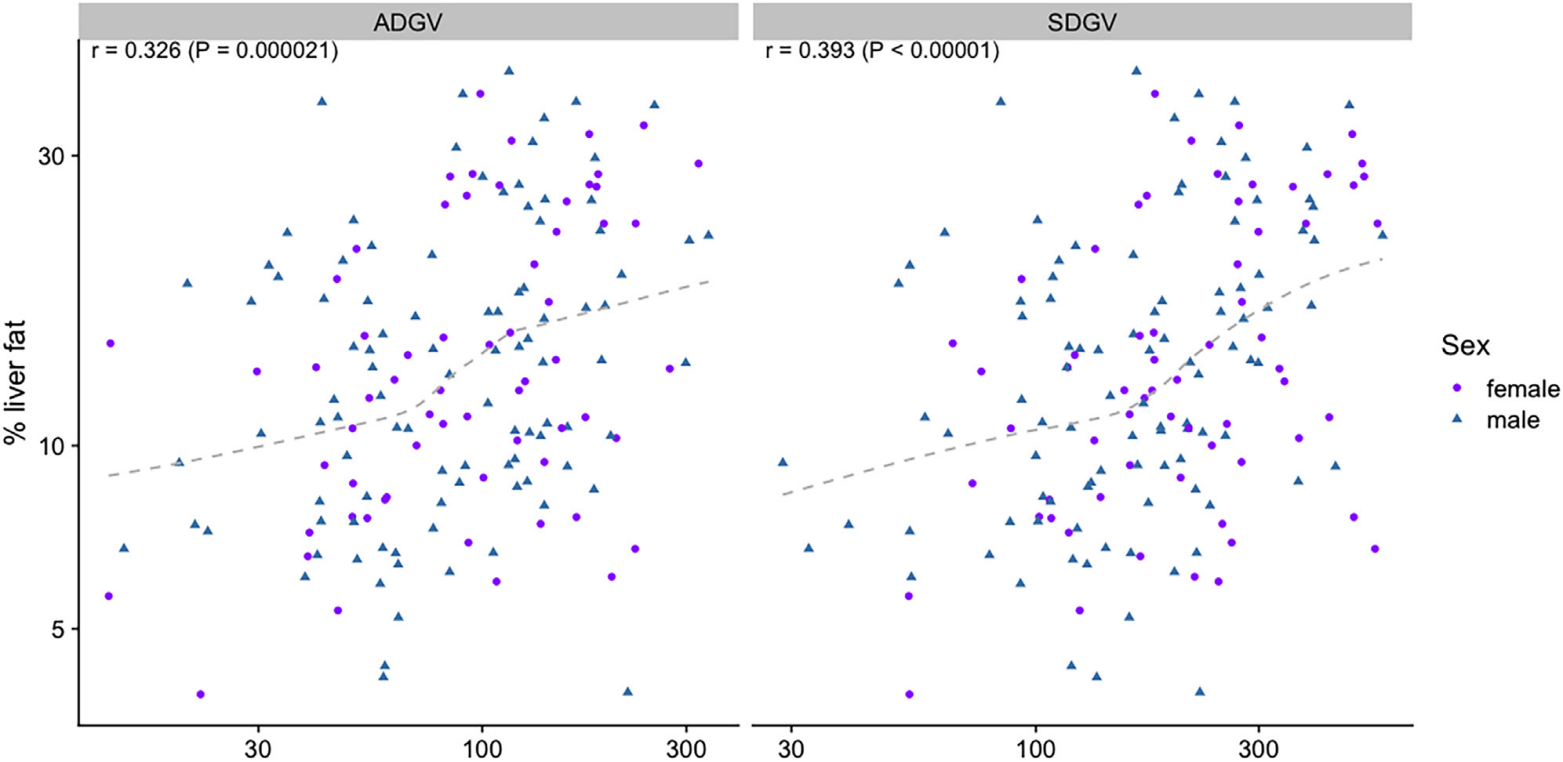
Liver fat versus the biomarkers ADGV and SDGV at baseline in the PLIS cohort The % liver fat and biomarkers are shown on log-scale.

First, we assessed the association of SDGV and ADGV (before any intervention) with liver fat content, calculated as % and measured with MRI in the PLIS cohort (Figure 1).

SDGV and ADGV levels correlated significantly (and similarly to FLI but stronger compared to other metabolites) with % of liver fat (Table 3).

**Table 3:** Pearson correlation and linear regression of SDGV, ADGV and other related markers with % of liver fat in the PLIS cohort at baseline

Biomarker	R	beta	*p* value	R^2^
SDGV	0.384	0.211	4.2e-07	0.148
ADGV	0.339	0.186	9.4e-06	0.115
FLI	0.453	0.249	<1e-08	0.206
GOCA	0.245	0.134	0.002	0.060
hArg	0.199	0.109	0.011	0.040
Arginine	−0.133	−0.073	0.089	0.018
BAIBA	−0.103	−0.056	0.194	0.011
ADMA	0.079	0.044	0.314	0.006
SDMA	0.012	0.006	0.881	1.4e-04

SDGV, symmetric α-keto-dimethylguanidinovaleric acid; ADGV, asymmetric α-keto-dimethylguanidinovaleric acid; FLI, fatty liver index; GOCA, 6-guanidino-2-oxocaproic acid; hArg, homoarginine; BAIBA, beta-aminoisobutyric acid; ADMA, asymmetric dimethylarginine; SDMA, symmetric dimethylarginine; R, correlation coefficient.

Next, we assessed the importance of SDGV and ADGV for diagnosing moderate to severe (i.e., >20% liver fat) steatosis. The 20% cut-off has been previously used for the evaluation of the diagnostic accuracy of other diagnostic tools (e.g., ultrasound) for liver fat.^8^ Here, a univariate logistic regression for different parameters showed that an elevated SDGV concentration is the strongest predictor for the presence of moderate to severe steatosis (2.965-fold higher risk for the presence of moderate-to severe steatosis by a one-standard deviation increase in the concentration of SDGV) (Table 4).

**Table 4:** Results of univariate logistic regression using standardized biomarkers on 1^st^ timepoint in the PLIS cohort

*Biomarker*	*OR*	*95%-CI*	*p-value*
**SDGV**	2.965	1.879–4.97	<0.0001
**FLI**	2.681	1.737–4.383	<0.0001
**ADGV**	2.438	1.593–3.927	<0.0001
**GOCA**	1.978	1.37–2.962	0.00021
**HomoArg**	1.314	0.918–1.917	0.13774
**Arginine**	0.826	0.579–1.175	0.28672
**BAIBA**	0.849	0.59–1.208	0.36352
**ADMA**	1.153	0.81–1.655	0.43088
**SDMA**	0.998	0.7–1.421	0.99097

SDGV: symmetric α-keto-dimethylguanidinovaleric acid; ADGV: asymmetric α-keto-dimethylguanidinovaleric acid; GOCA: 6-guanidino-2-oxocaproic acid; hArg: homoarginine; BAIBA: beta-aminoisobutyric acid; ADMA: asymmetric dimethylarginine; SDMA: symmetric dimethylarginine; beta: standardized estimated regression coefficient; OR: odds ratio.

**Figure 2:**
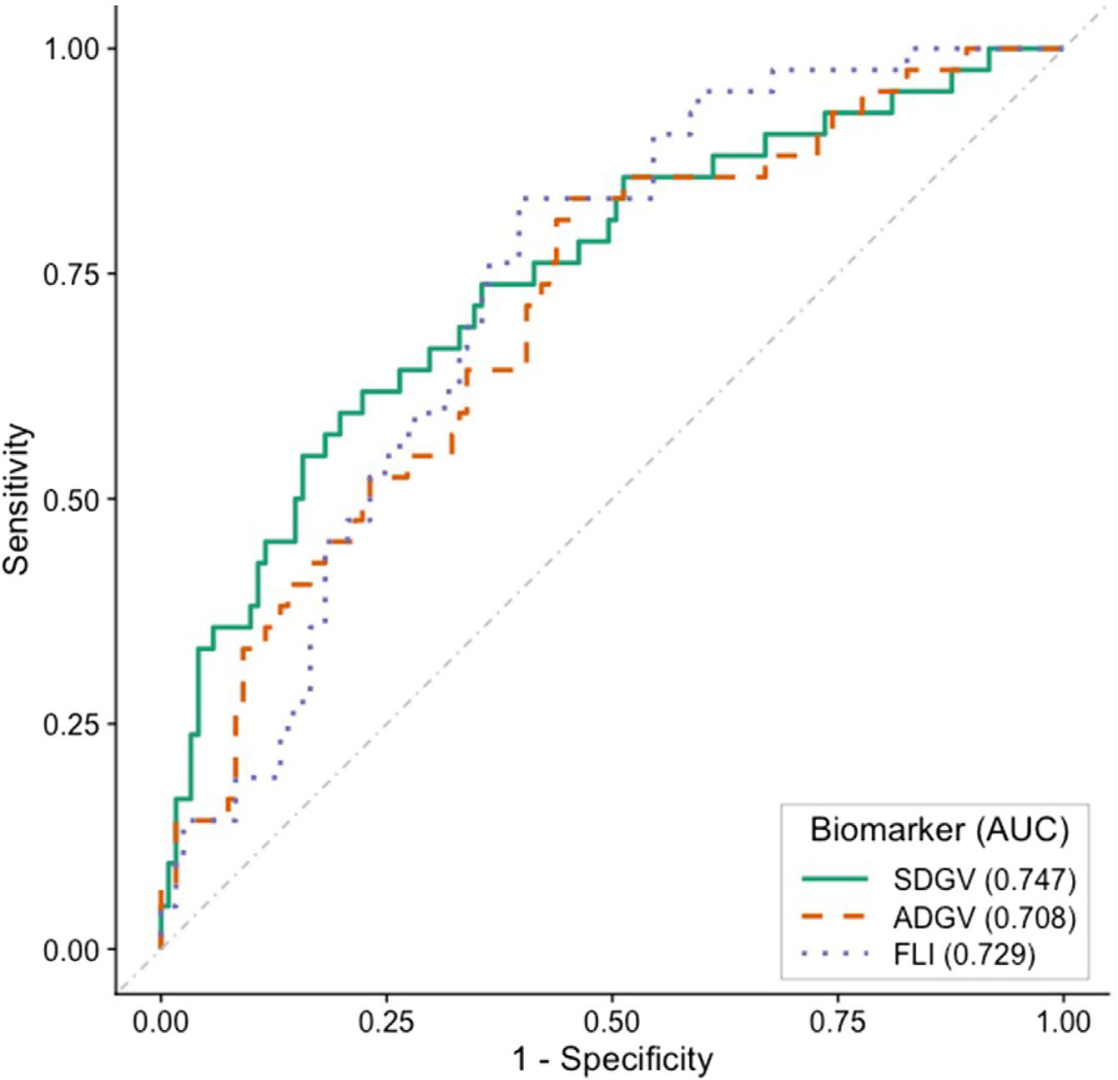
ROC curves at 20% liver fat threshold AUC: area under the curve; ADGV: asymmetric α-keto-dimethylguanidinovaleric acid; SDGV: symmetric α-keto-dimethylguanidinovaleric acid; FLI: fatty liver index.

At a threshold for liver fat detection of 20%, the area under the receiver operator characteristic curve (AUROC) for SGDV was 0.747 and for ADGV was 0.708, while that for FLI was 0.729 (Figure 2). When the threshold for liver fat detection was reduced to 10% in order to include also mild steatosis, SDGV and ADGV remained good predictive factors (AUROC of 0.683 and 0.663, respectively), but they were not superior to FLI (0.792). Thus, SDGV particularly, is a better predictor of moderate to severe steatosis than of mild steatosis.

The association of ADGV with liver fat was confirmed in the Sydney cohort consisting of biopsy-proven cases. Specifically, ADGV correlated significantly with steatosis grade based on liver histology (Table 5). Interestingly, ADGV concentrations were also positively associated with fibrosis stage. SDGV was not assessed in this cohort (see Discussion), with this being one of the limitations of the study.

**Table 5:** Correlation of AGTX2 metabolites with steatosis, portal inflammation and fibrosis in the Sydney cohort

Biomarker		Steatosis	Portal Inflammation	Fibrosis	NAS
**ADGV**	R	0.16	0.10	0.22	0.11
	Adjusted-P	3.6 x 10^−2^	0.23	2.6 x10^−3^	0.41
**BAIBA**	R	−0.09	−0.27	0.06	−0.16
	Adjusted-P	0.22	6.5 x 10^−4^	0.40	0.14
**Arginine**	R	−0.01	−0.09	−0.03	−0.073
	Adjusted-P	0.42	0.26	0.66	0.41
**Citrulline**	R	−0.03	−0.04	−0.02	−0.064
	Adjusted-P	0.74	0.67	0.83	0.41

ADGV: asymmetric α-keto-dimethylguanidinovaleric acid; BAIBA: beta-aminoisobutyric acid; R: correlation coefficient; adjusted –P: *p* value adjusted for age, sex, and body mass index. Steatosis, portal inflammation and fibrosis were assessed using a scoring system.

#### SDGV and ADGV track liver fat over time

**Figure 3:**
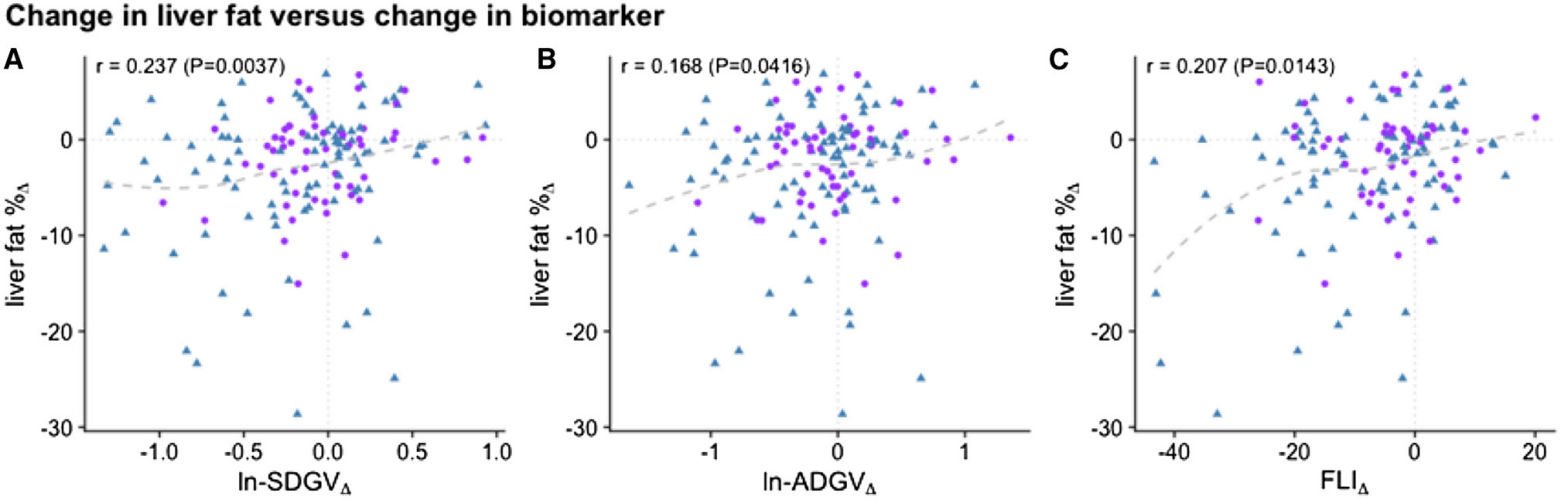
Change in biomarker vs. change in liver fat SDGV (A), ADGV (B), and FLI (C). Liver fat% determined by liver MRI; SDGV_lnΔ: change in symmetric dimethylguanidino valeric acid, log-scale; ALT_lnΔ: change in alanine aminotransferase, log-scale; r = correlation coefficient; P = *p* value.

We next compared “change in biomarker vs. change in liver fat”, for SDGV, for ADGV, as well as for FLI. Change in blood SDGV levels correlated better (r = 0.24, *p* = 0.0037) with change in % liver fat (Figure 3A) than did ADGV (r = 0.19, *p* = 0.042) (Figure 3B) or FLI (r = 0.21, *p* = 0.014) (Figure 3C).

The crude correlation of delta vs. delta has been criticized as a less rigorous test of biomarker performance due to the major confounder of “regression to the mean”: those with the greatest baseline liver fat have the greatest change in liver fat over time. Regression to the mean weighs in favor of the most affected individuals, and confounds assessment of the full spectrum of liver fat (mild to severe) simultaneously.

To address this problem, and to more accurately test liver fat “tracking” capability of each biomarker, we employed a linear mixed model that keeps the absolute level of biomarker and of liver fat independent from change in biomarker and liver fat. The results are plotted in Table 6, “Biomarker Change”. As the ultimate tracking biomarker would overcome regression to the mean (change in average biomarker, Table 6 “Biomarker Average” and deviation from the mean; Table 6, “Biomarker Change”), we assessed both. As seen in Table 6, only 3 blood biomarkers had significant tracking capability of liver fat change: SDGV, ADGV, and FLI. It is noteworthy but expected that ALT or AST were not associated with change in liver fat.

**Table 6:** Ability of biomarkers to track changes in liver fat over time

	*Biomarker Average*			*Biomarker Change*			Model Fit
Biomarker	Increase_avg_	Effect on FFW	P_avg_	Increase_chg_	Effect on FFW	P_chg_	R^2^_marg_
**SDGV**	+80.40%	+19.88%	<0.001	+12.75%	+02.66%	<0.001	0.1566
**ADGV**	+85.89%	+17.94%	<0.001	+13.88%	+02.40%	0.0034	0.1358
**ALT**	+53.73%	+24.59%	<0.001	+09.42%	+01.57%	0.0784	0.2139
**GOCA**	+84.04%	+13.69%	<0.001	+19.72%	+01.19%	0.1412	0.0884
**AST**	+32.31%	+13.09%	<0.001	+07.25%	+00.84%	0.3802	0.0829
**HomoArg**	+40.49%	+07.40%	0.051	+05.13%	+00.49%	0.5568	0.0437
**Arginin**	+18.53%	−04.26%	0.241	+03.05%	−01.17%	0.1537	0.0334
**ADMA**	+12.75%	+02.85%	0.436	+03.05%	+01.12%	0.1603	0.0293
**SDMA**	+20.92%	−02.22%	0.599	+03.05%	+00.12%	0.8896	0.0257

*p* values from likelihood ratio tests are reported (P_avg_ and P_chg_ for biomarker average and change); R^2^_marg_ corresponds to estimated marginal R^2^ i.e., the proportion of explained variability without the random patient-level effect.

SDGV: symmetric α-keto-dimethylguanidinovaleric acid; ADGV: asymmetric α-keto-dimethylguanidinovaleric acid; ALT: alanine transaminase; GOCA: 6-guanidino-2-oxocaproic acid; AST: aspartate transaminase; hArg: homoarginine; ADMA: asymmetric dimethylarginine; SDMA: symmetric dimethylarginine; FFW: intrahepatic lipids in % to the water signal; P = *p* value; R^2^ = correlation coefficient.

#### SDGV, not ADGV, is a faithful reporter of liver AGXT2 activity in a mouse model of fatty liver

**Figure 4:**
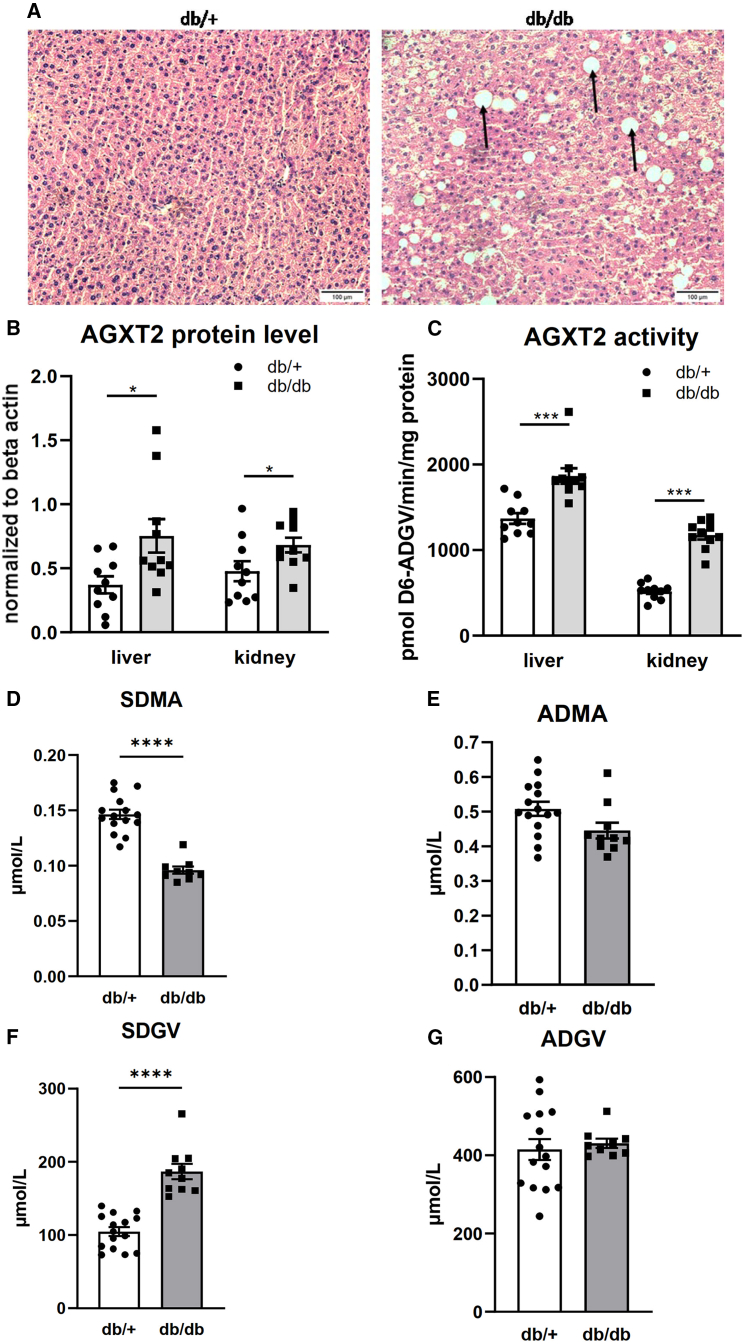
Findings in a mouse model of fatty liver (A) Representative images from hematoxylin-eosin-stained liver slides from db/db and db/+ mice. White spaces (arrows) indicate accumulation of hepatic fat. (B–G) (B) Protein level and (C) specific activity of alanine:glyoxylate aminotransferase 2 (AGXT2) in livers and kidneys of db/db and db/+ mice. Plasma levels of alanine:glyoxylate aminotransferase substrates (D) SDMA and (E) ADMA and products (F) SDGV and (G) ADGV in db/db and db/+ mice. D6-ADGV: stable-isotope labeled α-keto-dimethylguanidinovaleric acid; ADMA: asymmetric dimethylarginine; SDMA: symmetric dimethylarginine; ADGV: asymmetric α-keto-dimethylguanidinovaleric acid; SDGV: symmetric α-keto-dimethylguanidinovaleric acid. Data are represented as mean ± SEM and analyzed with unpaired two-sided Student’s t test. ∗*p* < 0.05; ∗∗∗*p* < 0.001; ∗∗∗∗*p* < 0.0001.

To investigate the mechanism behind the correlation of ADGV, SDGV, and liver fat, we used the db/db leptin-deficient mouse. 6-month-old db/db mice demonstrated excessive lipid accumulation in the liver in comparison to control db/+ animals (Figure 4A). The protein levels of the mitochondrial enzyme AGXT2, which produces ADGV from asymmetric dimethylarginine (ADMA) and SDGV from symmetric dimethylarginine (SDMA) were significantly higher in the db/db than in the db/+ mice (Figure 4B). Similarly, AGXT2 enzymatic activity was increased in db/db mice both in the liver and kidney (Figure 4C). In line with this data, when we measured the AGXT2 substrates and products in plasma, we discovered that the concentrations of SDMA (AGXT2 substrate) (Figure 4D) were significantly lower in the db/db than in the db/+ mice while concentrations of ADMA (AGXT2 substrate) were not decreased in db/db compared to db/+ mice (Figure 4E). As expected, the AGXT2 product SDGV was significantly increased in the plasma of db/db vs. db/+ mice (Figure 4F), whereas the AGXT2 product ADGV was not increased (Figure 4G).

## Discussion

Fatty liver disease due to systemic metabolic dysregulation affects at least a quarter of the population in most developed and developing countries. A major unmet need for its diagnosis at a population scale is availability of a simple diagnostic for liver fat quantification and for monitoring the amount of liver fat over time. While liver ultrasound and elastography techniques are commonly used, they are semi-quantitative at best, and because of the need for specialized equipment, are inadequate for population-level disease assessment and monitoring. Freely available non-invasive scores such as the fatty liver index, while useful for clinical research involving large patient cohorts, is infrequently used in daily practice and are at best a surrogate for semi-quantitative liver fat assessment. To our knowledge, measurement of SDGV in plasma is the first biomarker that correlates with the extent of liver fat, tracks liver fat extent over time, and performs equally well to the fatty liver index.

The principle findings of the current manuscript are that (1) plasma levels of SDGV are equal to, or more discriminatory, for liver fat than the fatty liver index; (2) plasma levels of SDGV have greater tracking ability for liver fat (as assessed by MRI) than the ALT and AST; and (3) the superior diagnostic performance of SDGV compared to ADGV likely reflects that SDGV is a faithful reporter of AGXT2 activity in the liver.

The current results interrogate the prognostic utility of a component metabolite of DMGV, i.e., SDGV—which demonstrates equal or superior discriminatory capacity to the fatty liver index. FLI is a well-known, widely accepted surrogate index of liver fat which was introduced in 2006 and is based on body mass index, waist circumference, plasma triglycerides, and gamma-glutamyl-transferase levels.^9^ An FLI <30 may be used to rule out fatty liver, while FLI ≥60 suggests presence of the disease. Previous studies have validated the usefulness of this index in predicting fatty liver^9^ making it commonly used for population based studies.^9^ However, the FLI is less frequently used in the clinic for individual patient management. This stems from a variety of factors including that waist circumference is infrequently measured and can be inaccurate if not undertaken by trained staff, is impacted by triglyceride lowering drugs, and because gamma glutamyl-transpeptidase (GGT) is impacted by liver injury and tracks more closely with oxidative stress. There is other published data demonstrating that the fatty liver index does not perform well enough to quantify the actual risk of fatty liver disease,^10^ does not accurately quantify steatosis, and is confounded by inflammation and fibrosis.^11^ Future prospective studies will be needed to assess the added diagnostic potential conferred by SDGV for the complications and outcomes of fatty liver disease.

DMGV, comprising both ADGV and SDGV, was previously shown by us and others to be an independent biomarker of fatty liver disease and an independent predictor of future diabetes over a decade in advance.^5^ DMGV is also a predictor of poor metabolic responses to exercise in middle-aged, overweight, sedentary individuals^12^ and correlates with poor metabolic response to exercise in young, fit, healthy, male soldiers.^13^ Further, DMGV is a reporter of unhealthy dietary intake—elevated by fat and sucrose intake and decreased by dietary fiber^14^; is significantly elevated in patients with type 2 diabetes, and correlates with vascular dysfunction, incident coronary artery disease, and cardiovascular mortality.^15^^,^^16^ A study by Najor et al. demonstrated that plasma DMGV levels are reduced after cardiopulmonary exercise testing in Framingham Heart Study participants.^17^ Despite this wealth of data, the precise molecular mechanisms by which DMGV and its metabolites track liver fat accumulation is at present not known.

In the current study, we provided a possible mechanism for elevated levels of SDGV for liver fat. Specifically, we show that db/db mice, an established model of hepatic steatosis and diabetes, have increased protein and specific activity levels of AGXT2 in the liver and kidney. Using histochemical staining the db/db mice had increased amounts of liver fat compared to control mice. AGXT2 has multiple enzymatic activities,^18^ with ADGV and SDGV being the products of catabolism of ADMA and SDMA, respectively. In line with increased protein levels and enzymatic activity, we demonstrated that levels of SDMA and homoarginine (substrates of AGXT2) were decreased in the plasma of db/db mice and that levels of the corresponding products (SDGV and 6-guanidino-2-oxocaproic acid) were increased. In contrast, no significant difference in the plasma levels of ADMA and ADGV was observed between the db/db and db/+ mice. An explanation could be that the levels of ADMA are regulated by proteolysis and metabolized by both AGXT2 and another enzyme, dimethylarginine dimethylaminohydrolase (DDAH).^19^ Conversely, SDGV is generated by single enzymatic conversion of SDMA by AGXT2. Therefore, the superior diagnostic performance of SDGV in the PLIS cohort is likely linked to its faithful reporting of AGXT2 activity. Of interest, a recent study in a human cohort revealed a likely pathogenic role for AGXT2 in fatty liver disease.^20^ This suggests that there is potential for SDGV to be more representative of pathology, in addition to being a biomarker.

Our study has several strengths, particularly, that SDGV was validated against the gold standard for liver fat quantification, namely MRI in the PLIS cohort. Further, the performance of SDGV longitudinally over 12 months to track liver fat indicates the robustness of this as a single biomarker. In the Sydney cohort, we confirmed that the DMGV pathway associates with liver fat.

In conclusion, we show that SDGV is an accurate marker of liver fat by comparison to the fatty liver index. As FLI is not used routinely in the initial screening or for tracking liver fat except in population based studies, our data suggests that SDGV should be further developed as a diagnostic for use, particularly in primary care. In combination with diagnostics for fibrosis assessment by tests such as ADAPT,^21^ proC3,^22^ and ,^23^ SDGV might be a useful addition for the assessment of fatty liver disease, for monitoring and for triage to specialist care.

### Limitations of the study

SDGV was not measured in the Sydney cohort because of unavailability of a commercial SDGV standard at the time of sample acquisition. We note that our data cannot be used to ascribe causality to AGXT2, ADGV, or SDGV. Therefore, in future it would be important the assess the ability of SDGV to track liver fat in other liver diseases associated with fat accumulation such as alcohol related liver disease and chronic hepatitis C, as also in patients with a mixed etiology for their liver disease. Additionally, in the animal experiments we used only male mice to generate the data.

## Resource availability

### Lead contact

Further information and request for resources and reagents should be directed to and will be fulfilled by the lead contact, Natalia Jarzebska (Natalia.Jarzebska@uniklinikum-dresden.de).

### Materials availability

This study did not generate new unique reagents.

### Data and code availability

• De-identified patients data have been deposited at Metabolomics Workbench. They are publicly available as of the date of publication. Accession number is listed in the key resources table.
• This paper does not report original code.
• Any additional information required to reanalyze the date reported in this paper is available from the lead contact upon request.

## Acknowledgments

JOS was funded by NSW Office of Medical Health and Research Early-Mid Career Researcher (212784) and Clinician-Scientist Awards, NHMRC-MRFF CVD grant (2024161), and a National Heart Foundation Vanguard (105595) and Future Leadership Fellowship (104853). The PLIS Study was supported by a grant (01GI0925) from the 10.13039/501100002347Federal Ministry of Education and Research (BMBF) to the German Center for Diabetes Research (DZD e.V.). ME and JG are supported by the Robert W. Storr Bequest to the Sydney Medical Foundation, University of Sydney; a 10.13039/501100000925National Health and Medical Research Council of Australia (NHMRC) Program Grant (APP1053206), Project, Ideas and Investigator grants (APP2001692, APP1107178, APP1108422, APP1196492) and a Cancer Institute, NSW grant (2021/ATRG2028).

## Author contributions

R.N.R. and J.F.O’S. were responsible for conceiving the study, supervision, interpretation of the results, and writing the manuscript. N.J. was responsible for performing the mice experiments, data analysis, and writing the manuscript. M.L. and M.K. were responsible for the statistical analysis of the data from the human cohorts. J.M.-L. was responsible for the measurements of the metabolites in the PLIS cohort and data interpretation. S.M.B.-B. was responsible for the study design, measurements of the metabolites in the PLIS plasma, data interpretation, and writing the manuscript. E.R. was responsible for performing mice experiments. S.R.B., A.G.M., and N.W. were responsible for writing the manuscript. P.S. and A.B. oversee the PLIS cohort, recruited the Dresden patients, and contributed to manuscript writing. J.G. oversees the Sydney cohort and contributed to manuscript writing. Y.C.K. performed the metabolomics in the Sydney cohort, data analysis, and contributed to manuscript writing. M.E. and F.W.C. recruited patients to the Sydney cohort, phenotyped patients, acquired blood samples, and contributed to data analysis. All authors approved the final version of the manuscript.

## Declaration of interests

The authors declare no competing interests.

## STAR★Methods

### Key resources table

**Table:** 

REAGENT or RESOURCE	SOURCE	IDENTIFIER
**Antibodies**		

Rabbit polyclonal anti-AGXT2	Sigma-Aldrich	Cat# HPA037382
Mouse monoclonal anti-beta actin, horseradish peroxidase conjugated	Sigma-Aldrich	Cat#A3854
Goat polyclonal anti-IgG (H + L)	Jackson ImmunoResearch	Cat#111-035-144

**Deposited data**		

Raw data	This paper	Metabolomics Workbench (Data Track ID 4940)

**Experimental models: Organisms/strains**		

Mouse BKS.Cg-*Dock7*^*m*^ +/+ *Lepr*^*db*^/J	Jackson Laboratory	JAX: 000642

**Software and algorithms**		

ImageJ	Schneider et al.^24^	https://imagej.nih.gov/ij/
Thermo Electron Xcalibur	ThermoFisher Scientific	https://www.thermofisher.com/order/catalog/product/de/en/OPTON-30965
Multi-QuantMD 3.0.2	Sciex	https://sciex.com/products/software/multiquant-software
R 4.1.1	Team, R. Core. R^25^	https://www.r-project.org/
GraphPad Prism	GraphPad	https://www.graphpad.com/

### Experimental model and study participant details

#### Description of the cohorts

##### PLIS cohort-dresden

The prediabetes lifestyle intervention study (PLIS) is a stratified-randomized, controlled multi-center trial involving eight study sites in Germany (ClinicalTrials.gov Identifier: NCT01947595). The primary hypothesis of the study is that individuals with prediabetes who are high-risk for failure to restore normal glucose regulation using conventional lifestyle intervention (LI) will benefit from an intensification of the LI. Prediabetes was diagnosed from fasting and 2-h post-challenge glucose levels after a standardized oral glucose tolerance test (OGTT), according to the criteria of the American Diabetes Association. Screening procedures also involved measurement of liver fat content, insulin sensitivity, and insulin secretion. For the current study, 213 participants from the Dresden site who had liver fat calculated as fat fraction (% liver fat) with MRI (Dixon method with IDEAL sequence^26^) determined at two timepoints 1 year apart were included. Briefly, the liver scan was performed using a single-breath-hold, 3D volume and a special 3-point proton density weighted Dixon technique (IDEAL IQ). The scan was based on a 6-echo planar imaging acquisition. The obtained fat-fraction maps were analyzed and all extraneous structured and any image artifacts were removed manually. A full description of the cohort is provided in.^27^ Blood samples were collected after an overnight fast, during the same visit as the MRI was performed. Informed consent was obtained from all the participants and the study was approved by the local authorities (EK 26012013). Due to ethical regulations, we did not share individual participants’ data.

##### Sydney cohort

This cohort consists of patients referred to a tertiary hepatology service on the basis of imaging evidence of steatosis (usually ultrasound), and/or abnormal plasma liver tests. Plasma samples from a total of 193 patients with biopsy-proven fatty liver disease were used in the analysis. The cohort was on average overweight with a BMI of 29.12 kg/m^2^; 24.4% had hypertension, 17.6% had diabetes mellitus, and 37.8% had dyslipidaemia. More than half the cohort had moderate to severe fibrosis (F2-4, 59.6%). Moderate to severe steatosis was present in the majority of the cohort (127 patients (65.8%)), and the majority had an activity score (NAS) of 4 or more (165 patients, 85.5%). Fibrosis, steatosis, portal inflammation, and NAS were assessed using a scoring system, where components are graded on a scale for steatosis (0–3), lobular inflammation (0–2), hepatocellular ballooning (0–2), and fibrosis (0–4). The NAS score is the sum of scores for steatosis, inflammation and ballooning, and does not include the stage of fibrosis. Informed consent was obtained from all the participants and the study was approved by the local authorities (2000/8/4.14 (1061)). Due to ethical regulations, we did not share individual participants’ data.

##### Murine model

All animal experiments were approved by authorities from the Technische Universität Dresden (permission number: TVT 2/2019). Six-month-old db/db (*n* = 10) and db/+ (*n* = 15) male mice (the Jackson Laboratory BKS.Cg-*Dock7*^*m*^ +/+ *Lepr*^*db*^/J; stock no. 000642) were used for the experiments. All animals were housed at constant humidity (60 ± 5%), temperature (24 ± 1°C), and a 12 h light/dark cycle (6a.m. to 6p.m. light). Mice had unlimited access to water and food (standard rodent chow).

### Method details

#### Metabolite measurements in PLIS cohort and mice

Levels of the AGXT2 substrates ADMA, SDMA, beta-aminoisobutyric acid (BAIBA), L-homoarginine and L-arginine and the products ADGV, SDGV and 6-guanidino-2-oxocaproic acid (GOCA) were measured in plasma of the PLIS cohort patients and in mouse plasma by isotope-dilution tandem mass spectrometry (LC-MS/MS) as previously described.^28^^,^^29^ Briefly, for the measurement of homoarginine, the sample preparation consisted only of the addition of the stable isotope-labeled internal standard D_4_-L-homoarginine and protein precipitation. The analytes were separated isocratically on a Atlantis HILIC silica column with 5 μm particle size and with the dimentions 150 × 2.1 mm (Waters, Eschborn, Germany). Detection took place by a Thermo Fisher Scientific (Waltham, MA, USA) TSQ Discovery May triple quadrupole mass spectrometer. System control and data handling were carried out by the Thermo Electron Xcalibur software, version 1.2). The calibration function was linear in the range of 0.1–10 μmol L^−1^. The intra-day precision better than 4% RDS in plasma. The accuracy was always better than 5% deviations. ADMA and SDMA were detected by ESI MS/MS, providing high selectivity and sensitivity. The calibration functions were linear in the ranges of 0.15–3 μmol/L for ADMA and 0.2–4 μmol/L for SDMA. The method employed D(7)-ADMA and D(6)-SDMA as internal standards. Matrix independency and a high intra-day precision of 2.12% for ADMA and 2.83% for SDMA were achieved. The respective inter-day precision values were 3.77% for ADMA and 3.86% for SDMA.

#### Metabolite measurement in sydney cohort

Levels of ADGV, BAIBA, Arginine, and Citrulline were measured using liquid chromatography–tandem mass spectrometry (LC-MS/MS) on an Agilent 1260 Infinity HPLC System (Agilent Technologies, Santa Clara, CA, USA) coupled to an AB SCIEX QTRAPVR 5500 MS tandem mass spectrometer (MS/MS) triple quadrupole (QqQ) mass analyzer operating in MRM scan mode in positive ion mode using an AtlantisVR HILIC column. Samples were randomized and an internal pooled sample was run every 10 samples for quality control. All raw data files (Analyst software, version 1.6.2; AB Sciex, Foster City, CA, USA) were imported into Multi-QuantMD 3.0.2 Software for MRM Q1/Q3 peak integration. To account for any performance drift in the LC-MS/MS, the metabolite abundance in each sample was normalized to the bookended pooled plasma sample (every 10 samples), deriving a ‘Normalized area (AU)’ (normalized abundance) for each metabolite per standard practice.

#### Collection of plasma and organs

Mice were subjected to isofluorane anesthesia and blood was collected by cardiac puncture into EDTA containing tubes (final concentration 5 mmol/L). Mice were subsequently perfused with 0.9% (w/v) NaCl solution. Liver and kidneys were harvested, flash-frozen and stored at −80°C until further analysis. Plasma was separated by centrifugation and stored at −80°C.

#### Histochemistry

Immediately after isolation, liver samples were fixed in cold 4% paraformaldehyde diluted in phosphate-buffered saline at 4°C and processed for paraffin embedding, and cross-sectioned to obtain 4 μm-thick sections and mounted on glass slides. Tissue sections were deparaffinized in xylene 3 × 5 min and rehydrated in descending concentrations of ethanol (100%, 96%, 70% and 40%, 2 min each). The slides were placed in hematoxylin solution for 10 min, rinsed with running tap water for 10 min and immersed in distilled water for 1 min. Next, the slides were put in eosin solution for 2 min, rinsed three times in distilled water, dehydrated in ascending concentrations of ethanol (40%, 70%, 96% and 100%, 2 min each), cleared in xylene 3 × 2 min and mounted with DePeX medium. Photomicrographs were taken with a Zeiss Apotome microscope (Germany) and analyzed in ImageJ,^24^ version 1.53 c (National Institute of Health).

#### Detection of AGXT2 protein levels and activity

The two isoforms of DMGV, namely ADGV and SDGV, are the product of the conversion of substrates asymmetric dimethylarginine (ADMA) and symmetric dimethylarginine (SDMA) by the enzyme AGXT2, which resides principally in hepatocytes and renal tubular epithelial cells,^30^ see Supplemental Table 2 for an illustration of all relevant substrates and products of AGXT2, as measured in this manuscript. Detection of AGXT2 protein in kidney and liver lysates from db/db mice was performed by immunoblotting using rabbit anti-AGXT2 antibody (Sigma-Aldrich #HPA037382, dilution 1:2000), which recognizes both mouse and human AGXT2 using a previously established protocol.^30^ Briefly, tissue homogenates (15 μg of total protein) were prepared and diluted with Laemmli buffer (62 mM Tris–HCl pH 6.8, 2% SDS, 10% glycerol, 0.01% bromophenol blue, and 0.4 mM dithiothreitol). After incubation at 95°C for 5 min, proteins were separated by SDS–PAGE under reducing conditions on 10% polyacrylamide gels and transferred to nitrocellulose membranes (Protran Nitrocellulose Transfer Membrane; Whatman, Dassel, Germany) using a tank blotting system from Bio-Rad (Munich, Germany). Membranes were stained by Ponceau reagent (Sigma-Aldrich, St. Louis, MI, USA) to control for the protein transfer and then blocked in 5% milk for 1 h at 37°C. Membranes were incubated overnight at 4°C with a primary antibody, washed three times in TBST (50 mM Tris–HCl pH 7.5, 150 mM NaCl, 0.1% Tween 20) and then incubated with a 1:10,000 horseradish peroxidase-conjugated goat anti-rabbit antibody (Jackson ImmunoResearch, Catalog #111-035-144) for 2 h at room temperature (RT). Immunoreactive bands were visualized using Roche Lumi-Light Western Blotting Substrate (Roche Diagnostics, Mannheim, Germany). To control for sample loading, membranes were incubated with 1:5000 horseradish peroxidase-conjugated polyclonal mouse anti-beta actin antibodies (Sigma-Aldrich, Catalog #A3854) for 2 h at RT. Blotted proteins were detected using a PeqLab Fusion Fx7 Imaging System (PeqLab, Erlangen, Germany).

Measurement of AGXT2 activity in tissues from db/db mice was performed using stable isotope-labeled ADMA as substrate with subsequent detection of labeled ADGV as previously described.^31^ Briefly, app. 20 mg frozen sample of kidney or liver was cut and put into ice-cold lysis buffer (Mammalian cell lysis kit, Sigma-Aldrich, Munich, Germany). Tissue was disrupted, the mixture was left on ice for 20 min and centrifuged for 10 min at 4 °C at 11 000 x g. AGXT2 activity was measured in 100 mmol phosphate buffer at pH 8 with the addition of 0.5 mmol/L D6-ADMA. The incubation was carried on for 1 h at 37°C and stopped by adding 20 μL 5 mol/L perchloric acid. The metabolites were detected using HPLC-tandem mass spectrometry with internal standards.

### Quantification and statistical analysis

Prior to statistical modeling, we preprocessed both the biomarkers and liver fat measurements by applying a logarithm to remedy their right-skewness. The biomarkers on log-scale were then mean-centered and scaled to have unit variance for better comparability between the biomarkers. Linear mixed models were used to quantify the relationship between a biomarker and liver fat as a dependent variable. Each biomarker was measured twice, and we assessed the effect of both its average and its change over time per patient. A random intercept effect at patient level accommodates the correlation of the repeated liver fat measurements per patient. We checked the model assumptions by means of diagnostic plots (residual plots, QQ-plots) for each regression model. Statistical significance of a predictor in the model was determined by likelihood ratio tests. For sensitivity, specificity, positive predictive value, and negative predictive value, a cut-off level of MRI defined-fat free water (FFW) was used as originally described by.^32^ All the data was quantified in a blinded manner. Statistical significance was determined as *p* < 0.05. The data analysis for the Sydney was performed using R (4.1.1)^25^ and the limma (3.49.1) package was used to perform the association analysis. The data for the PLIS cohort was analyzed using R (4.3.2)^25^ and the linear mixed model was done with the help of the R-package glmmTMB (1.1.8). The mice data was tested for normality using the Shapiro-Wilk test and analyzed by unpaired two-sided Student’s t test and the analysis was done in GraphPad Prism 9.1.2. The graphs on Figure 4 represent mean ± SEM. The n number for Figures 4B and 4C is 10 (number of mice) and for Figures 4D–4G 15 for db+ groups (number of mice) and 10 for db/db groups (number of mice).
