# Supplementary material for: Symmetric dimethylguanidino valeric acid, a novel single biomarker of hepatic steatosis
Source: iScience. 2024 Nov 13;27(12):111366. doi: 10.1016/j.isci.2024.111366 (PMC11629207; doi:10.1016/j.isci.2024.111366)
Supplement: Document S1. Figures S1, S2 and Table S1 [file mmc1.pdf]

iScience, Volume *27*

Supplemental information

Symmetric dimethylguanidino valeric acid, a novel single biomarker of hepatic steatosis

Roman N. Rodionov, Natalia Jarzebska, Yen Chin Koay, Mengbo Li, Matthias Kuhn, Stefan R. Bornstein, Jens Martens-Lobenhoffer, Mohammad Eslam, Fei Wen Chen, Elena Rubets, Alexander G. Markov, Norbert Weiss, Andreas Birkenfeld, Peter Schwarz, Stefanie M. Bode-Böger, Nikolaos Perakakis, John F. O'Sullivan, and Jacob George

Supplemental Data

Supplemental Table 1: Results of univariate logistic regression using standardized biomarkers on 1st time point only at the 10% threshold of liver fat. Reported odds ratio (OR) is for an increase by one standard deviation in the biomarker. Row order is determined by P-value.

Biomarker	OR	95%-CI	P-value
FLI	3.346	2.234 - 5.262	< 0.0001
SDGV	1.951	1.374 - 2.855	0.00014
ADGV	1.834	1.3 - 2.655	0.00046
HomoArg	1.459	1.046 - 2.066	0.02608
GOCA	1.486	1.046 - 2.179	0.02651
Arginine	0.692	0.48 - 0.974	0.03432
BAIBA	0.765	0.542 - 1.068	0.11637
ADMA	1.155	0.831 - 1.616	0.39016
SDMA	0.921	0.661 - 1.281	0.62509

SDGV: symmetric α-keto-dimethylguanidinovaleric acid; ALT: alanine transaminase; FLI: fatty liver index; ADGV: asymmetric α-keto-dimethylguanidinovaleric acid; GOCA: 6-guanidino-2-oxocaproic acid; AST: aspartate aminotransferase; hArg: homoarginine; BAIBA: beta-aminoisobutyric acid; ADMA: asymmetric dimethylarginine; SDMA: symmetric dimethylarginine; beta: standardized estimated regression coefficient; OR: odds ratio.

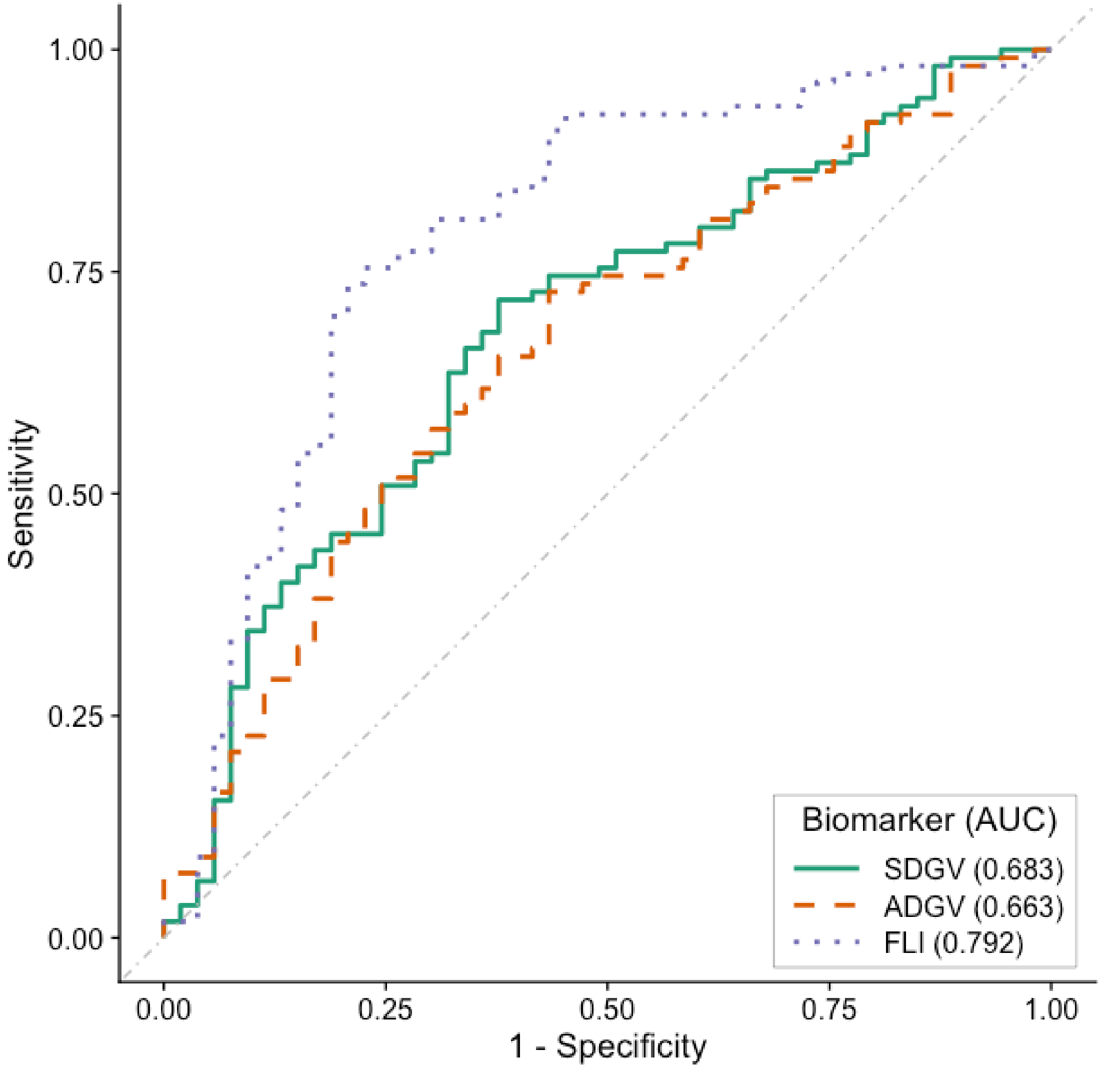

Supplemental Figure 1: ROC curves at 10% liver fat threshold. AUC: area under the curve; ADGV: asymmetric α-keto-dimethylguanidinovaleric acid; SDGV: symmetric α-keto-dimethylguanidinovaleric acid; FLI: fatty liver index; ALT: alanine transaminase.

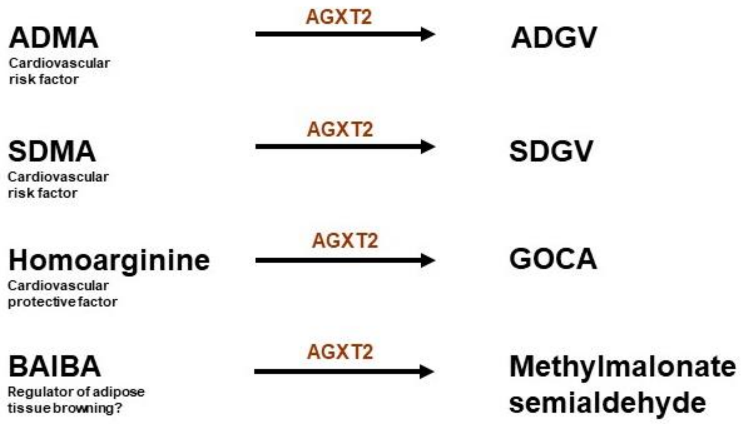

Supplemental Figure 2: Illustration of substrates and products of AGXT2. ADMA: asymmetric dimethylarginine; ADGV: asymmetric α-keto-dimethylguanidinovaleric acid; SDMA: symmetric dimethylarginine; SDGV: symmetric α-keto-dimethylguanidinovaleric acid; GOCA (6-guanidino-2-oxocaproic acid); BAIBA: beta-aminoisobutyric acid.
